# Why It Is Not Always Anxiety: A Tough Diagnosis of Stiff Person Syndrome

**DOI:** 10.1155/2017/7431092

**Published:** 2017-08-14

**Authors:** Carmen Elena Cervantes, Hsien Lee Lau, Tina Ataian Binazir, Keith O. O'Brien, Jonathan S. Cross

**Affiliations:** Department of Medicine, Aventura Hospital and Medical Center, Aventura, FL, USA

## Abstract

Anxiety disorder is a commonly used diagnosis that may mask underlying conditions. Stiff person syndrome (SPS) is a rare neuroimmunological disorder characterized by progressive rigidity and painful muscle spasms affecting axial and lower extremity musculature. These episodes can be triggered by sudden movement, noise, or emotional stress, which may present as a psychiatric condition. We report the case of a 30-year-old female who presented with recurrent panic attacks with multiple prior hospital admissions for anxiety, rigidity, and difficulty in walking. Previous electroencephalogram (EEG) and brain and cervical spine magnetic resonance imaging (MRI) were unremarkable. She was empirically treated with diazepam and beta-blockers for SPS, which was confirmed by positive glutamic acid decarboxylase (GAD) antibodies. The patient's symptoms became refractory to benzodiazepines and required steroids with intravenous immunoglobulin (IVIG). Her rigidity subsequently responded to plasmapheresis. In SPS, antibodies in the cerebrospinal fluid (CSF) most commonly target the GAD antigen on gamma-aminobutyric acid (GABA) neurons. The goal of treatment is to ameliorate symptoms and improve quality of life. Our case of SPS was masked as generalized anxiety disorder for at least six years since onset of symptoms. The criteria for both diagnoses may overlap as seen in this patient.

## 1. Introduction

Anxiety disorder is a commonly used diagnosis that may mask underlying conditions. Anxiety is mostly associated with rare diseases and is frequent among patients of some socioeconomic and cultural backgrounds. Stiff person syndrome (SPS) is a rare neuroimmunological disorder identified as an autoimmune or paraneoplastic syndrome [1]. It is characterized by progressive rigidity and painful muscle spasms affecting axial and lower extremity musculature. These episodes can be triggered by sudden movement, noise, or emotional stress, which may present as a psychiatric condition. Other cardinal symptoms include paroxysmal autonomic dysfunction with associated diaphoresis, tachycardia, pupillary dilatation, and hypertension [1–3]. Here, we report the case of a young patient previously diagnosed with anxiety disorder in whom years later SPS was identified.

A 30-year-old Haitian female with a past medical history of anxiety disorder and baseline tachycardia presented with recurrent panic attacks. The patient reported anxiety, palpitations, muscle stiffness, and painful spasms which started in the pelvis and radiated to bilateral lower extremities. Within a two-year period, she had multiple admissions for similar episodes of rigidity and difficulty in walking resulting in a diagnosis of anxiety. However, her first symptoms were reported six years previously. Prior to admission, she was empirically treated with diazepam and beta-blockers for SPS, while lab tests were pending.

On examination, vital signs were stable. Neurological exam revealed dystonia and tremors which were most apparent in bilateral lower extremities. Spasms were noted in all four limbs, as well as increased deep tendon reflexes: 3+ hyperreflexia in biceps, triceps, brachioradialis, and patellar and Achilles tendons. Cardiac and respiratory exams were unremarkable.

During previous hospital admissions, EEG did not detect ictal activity. Brain and cervical spine MRIs were unremarkable. Tests measuring creatinine kinase, aldolase, toxicology, and thyroid function were within normal ranges. The patient's GAD antibodies were significantly elevated showing a level above 250 (normal: 0–5) units/mL.

The patient was initially treated with intravenous steroids and diazepam followed by IVIG for 3 days and finally improved with 5 cycles of plasmapheresis. The patient was discharged after 6 weeks with maintenance therapy of oral baclofen and diazepam.

## 2. Discussion

The distinguishing feature of our case is the presentation of SPS, concealed as an anxiety disorder. Stiff person syndrome is rare, with an estimated incidence of 1 per one million. It affects women 2-3 times more than men with an average age of presentation of 20–50 [1–3, 6]. The duration from symptom onset to diagnosis ranges from 1 to 18 years, with a mean of 6.2 years [9].

The SPS diagnostic criteria are outlined in Table 1. Clinical manifestations have been categorized as classic SPS or variant SPS, such as stiff limb syndrome and progressive encephalomyelitis [2, 6, 9]. The stiffness and rigidity in classic SPS can be triggered by minimal physical or emotional stimuli, overtime causing increased anxiety and severe disability [6].

In SPS, antibodies in the CSF commonly target the GAD antigen on gamma-aminobutyric acid (GABA) neurons [1]. However, anti-GAD antibodies are present only in two-thirds of patients [6, 9]. GABA neurons inhibit spontaneous discharges from spinal motor neurons [1]. Therefore, their absence leads to a continuous activation of muscle motor units. It is unclear whether the disease process underlying SPS is directly responsible for anxiety disorders or whether anxiety disorders develop as a reaction to disability associated with SPS. A recent neuropsychological cohort study of 10 patients with SPS and symptoms of anxiety suggested that anxiety was a reaction to disability. Low levels of GABA lead to an overall higher firing frequency of nerve cells, which can manifest as either anxiety or even seizure disorders. This may lower the threshold for overactivity of the amygdala, the fear and anxiety center of the brain [10].

When analyzed, criteria for both disorders may overlap as represented in Table 1. The text in italics specifically shows which conditions our patient met from both diseases. She met all criteria for anxiety disorder, except for the fifth component: the underlying medical condition which was not initially identified. For SPS, she met all the conditions, except for EMG which was not performed. With regard to demographics, she fits two features of both conditions: age group of 20–50 years and female gender.

The goal of treatment is to improve quality of life by providing symptom relief. Benzodiazepines are considered first-line treatment in SPS [8, 10]. As a GABA_A_ agonist, diazepam is used for its muscle relaxant and anxiolytic properties. Baclofen is used orally with diazepam for its GABA_B_ agonist activity to manage spasticity [1, 6, 8].  Table 2 outlines more treatment options.

This case yields insight into the similarities between SPS and anxiety which may delay diagnosis and management for an internist or general neurologist. Anxiety and functional disorders presenting with recurrent muscular rigidity and/or episodic autonomic symptoms resembling panic attacks should raise alarm of an alternative neurologic disease. Eating disorders, hysterical paralysis, phobias, posttraumatic stress disorder, and agoraphobia have been misdiagnosed when SPS was the underlying condition [11]. Psychogenic movement disorder is the most important differential diagnosis of SPS. It is characterized by a normal neurological examination (including a normal blink reflex test), distractibility, and symptom improvement without significant interventions [12]. Hysterical paralysis and other conversion disorders rarely persist for more than 1 year, while SPS will have a more progressive course [13, 14]. By continuing to broaden our understanding and knowledge of the diverse presentations of SPS, we can decrease the morbidity associated with long-standing misdiagnosis.

## Figures and Tables

**Table 1:** Diagnostic criteria for generalized anxiety disorder compared to Dalakas criteria for SPS.

Generalized anxiety disorder criteria [1]	Dalakas criteria for SPS [4]
(1) *Excessive anxiety and worry*, occurring more days than not for *at least 6 months*.	(1) *Stiffness in the axial muscles*, particularly abdominal and thoracolumbar paraspinal muscles, leading to a fixed deformity: hyperlordosis. (2) Superimposed painful spasms *precipitated by unexpected noises, emotional stress, or tactile stimuli*. (3) Confirmation by electromyography (EMG). (4) *Absence of neurological or cognitive impairment *that could explain the stiffness. (5) *Positive serology: GAD* or amphiphysin autoantibodies. (6) *Response to diazepam*.
(2) The individual finds it *difficult to control the worry*.	
(3) The anxiety and worry are associated with *three* or more of the following:	
(a) *Restlessness and feeling keyed up or on edge*	
(b) *Being easily fatigued*	
(c) Difficulty concentrating or mind going blank	
(d) Irritability	
(e) *Muscle tension*	
(f) Sleep disturbances	
(4) *Symptoms cause significant distress or impairment in social, occupational, or other areas of functioning*.	
(5) *Symptoms are not attributable to the physiological effects of a substance or another medical condition*.	
(6) *Symptoms are not explained by another mental disorder*.	

(1) Table adapted from DSM-V criteria for generalized anxiety disorder [5]. (2) Table adapted from Dalakas criteria for SPS [6]. *Note*. Text in italic represents symptoms/signs for the patient, meeting criteria for both GAD and SPS.

**Table 2:** Treatment options for stiff person syndrome.

Type of treatment	Treatment description
*First-line treatment*	(i) Benzodiazepines (ii) Diazepam is used both as an anticonvulsant and as a muscle relaxant, with oral baclofen
*Other GABAergic drugs*	(i) Gabapentin, tiagabine, and levetiracetam have been used for reduction in symptoms
*Oral *versus* intrathecal baclofen*	(i) Oral baclofen with diazepam is part of the first-line treatment for GABA_B_ agonist activity directed against spasticity (ii) Intrathecal baclofen has been used for severe spasticity and has shown significant improvement
*Treatment with intravenous immunoglobulin *versus* plasmapheresis *	(i) IVIG (2 g/kg over two to five days) is used for symptomatic relief when first-line treatment fails and patients have severe disability in carrying out daily activities (ii) Plasmapheresis showed promising results in 56% of patients when first-line therapy failed [7]
*Rituximab*	(i) Monoclonal antibody that binds to the B-lymphocyte cluster of differentiation surface antigen (ii) Administration: 2 doses are infused with spacing of 7–14 days or as four weekly infusions (iii) Improves gait when benzodiazepines and monthly IVIG treatment have failed

Modified from Ariño et al. [8].
